# Supplementary material for: A lateralized sensory signaling pathway mediates context-dependent olfactory plasticity in C. elegans
Source: bioRxiv. 2025 Jul 29:2025.07.25.666858. Preprint. [Version 1] doi: 10.1101/2025.07.25.666858 (PMC12324235; doi:10.1101/2025.07.25.666858)
Supplement: Supplement 1 [file NIHPP2025.07.25.666858v1-supplement-1.pdf]

bioRxiv preprint doi: https://doi.org/10.1101/2025.07.25.666858; this version posted July 29, 2025. The copyright holder for this preprint (which was not certified by peer review) is the author/funder, who has granted bioRxiv a license to display the preprint in perpetuity. It is made available under aCC-BY 4.0 International license.

S1 Data

Numerical data for all behavioral and calcium imaging experiments are provided at https://doi.org/10.5281/zenodo.15939087.

bioRxiv preprint doi: https://doi.org/10.1101/2025.07.25.666858; this version posted July 29, 2025. The copyright holder for this preprint (which was not certified by peer review) is the author/funder, who has granted bioRxiv a license to display the preprint in perpetuity. It is made available under aCC-BY 4.0 International license.

SUPPLEMENTARY FIGURE LEGENDS

Fig S1. AWC-expressed guanylyl cyclase genes.

A) Expression levels of indicated receptor and soluble guanylyl cyclase genes in the two AWC neurons from CeNGEN [33]. Expression values are in transcripts per million (TPM) without thresholding (www.cengen.org) [33]. Receptor guanylyl cyclases are bolded.

B) Gene structure of *gcy-12* and the encoded *gcy-12.a* and *gcy-12.b* transcript isoforms. The molecular lesions in the *ks100* and *nj10* alleles are indicated [43].

C) Behavioral responses of wild-type and *gcy-12(ks100)* animals to a point source of 10^{-3} IAA in 10^{-4} IAA as the saturating odorant. Each dot is the chemotaxis index calculated from a single assay of ~50-100 animals. Data shown are from at least three independent days. Horizontal and vertical bars are the mean and SEM, respectively. *: different from wild-type at $P<0.05$ (unpaired t-test with a post-hoc Welch's correction).

S2 Fig. Mutations in *gcy-12* affect context-dependent hexanol response plasticity only in AWC^{OFF}.

A-C) (Left) Average changes in GCaMP fluorescence in AWC (A, B) or ASH (C) neurons in wild-type and *gcy-12* mutants upon addition of a 30 sec pulse of 10^{-4} hexanol (A, C) or IAA (B) indicated by a short solid line in 10^{-4} concentration of sIAA (long solid gray line). Shaded regions are SEM. (Middle) Heatmaps of changes in fluorescence intensity corresponding to the responses shown at left in each panel. Each row in the heatmaps shows responses from a single neuron. (Right) Quantification of peak changes in fluorescence intensity upon odorant addition and/or removal in each panel. Each circle is the value from a single neuron. Horizontal and vertical bars indicate the mean and SEM, respectively. *, **, and ***: different from

bioRxiv preprint doi: https://doi.org/10.1101/2025.07.25.666858; this version posted July 29, 2025. The copyright holder for this preprint (which was not certified by peer review) is the author/funder, who has granted bioRxiv a license to display the preprint in perpetuity. It is made available under aCC-BY 4.0 International license.

corresponding wild-type at P<0.05, 0.01 and 0.001, respectively (A: Kruskal-Wallis with Dunn's post-hoc correction; B, C: Mann-Whitney-Wilcoxon test); ns: not significant. Data in A are repeated in Fig 2C, separated by AWC^{OFF} and AWC^{ON} neurons.

S3 Fig. *gcy-12* mutants exhibit asymmetric hexanol-evoked calcium responses in sIAA in AWC neuronal cilia.

A-C) (Left) Average changes in GCaMP fluorescence in the cilia of AWC neurons in wild-type and *gcy-12* mutants upon addition of a 30 sec pulse of 10^{-4} hexanol (A) or IAA (B) indicated by a short solid line, and 10^{-4} hexanol in 10^{-4} concentration of sIAA (C, long solid gray line). Shaded regions are SEM. (Middle) Heatmaps of changes in fluorescence intensity corresponding to the responses shown at left in each panel. Each row in the heatmaps shows responses from a single neuron. (Right) Quantification of peak changes in fluorescence intensity upon odorant addition or removal in each panel. Each circle is the value from a single neuron. Horizontal and vertical bars indicate the mean and SEM, respectively. ** and ***: different from corresponding wild-type at P<0.01 and 0.001, respectively (A, C: Mann-Whitney-Wilcoxon test, B: unpaired t-test with a post-hoc Welch's correction n); ns: not significant.

S4 Fig. Context-dependent plasticity defects in *gcy-12* mutants are partly odorant-specific.

A-D) Heatmaps of changes in fluorescence intensity corresponding to the responses shown in Fig 3B, 3C, 3E and 3F. Each row in the heatmaps shows responses from a single AWC^{OFF} or AWC^{ON} neuron from different animals.

bioRxiv preprint doi: https://doi.org/10.1101/2025.07.25.666858; this version posted July 29, 2025. The copyright holder for this preprint (which was not certified by peer review) is the author/funder, who has granted bioRxiv a license to display the preprint in perpetuity. It is made available under aCC-BY 4.0 International license.

S5 Fig. Loss of *gcy-12* only in AWC^{OFF} is not sufficient to alter hexanol response plasticity in sIAA.

A) Genomic structure of *gcy-12* indicating the sites of insertion of fluorescent reporter sequences at the endogenous locus. Also see S1B Fig.

B) Representative images showing expression of the two reporter-tagged *gcy-12* isoforms in the head. Arrows indicate putative neuronal soma. Expression of *gcy-12.b* was undetectable. Anterior at left. Scale bar: 5 µm.

S6 Fig. Loss of AWC fate asymmetry abolishes asymmetry in the odorant response defects of *gcy-12* mutants.

A-F) Heatmaps of changes in fluorescence intensity corresponding to the responses shown in Fig 5A-F. Each row in the heatmaps shows responses from a single AWC^{OFF} or AWC^{ON} neuron from different animals. Both AWC neurons are considered to be AWC^{OFF} in *nsy-5* and *nsy-5; gcy-12* mutants; both AWC neurons are considered to be AWC^{ON} in *nsy-1* and *gcy-12 nsy-1* mutants. *gcy-12* data are repeated from Fig 2 and indicated in light pink.

bioRxiv preprint doi: https://doi.org/10.1101/2025.07.25.666858; this version posted July 29, 2025. The copyright holder for this preprint (which was not certified by peer review) is the author/funder, who has granted bioRxiv a license to display the preprint in perpetuity. It is made available under aCC-BY 4.0 International license.

S1 Video. Intracellular calcium levels in AWC^{ON} are increased upon addition of hexanol in both wild-type and *gcy-12* mutants.

Fluorescence changes in AWC^{ON} in wild-type (left) and *gcy-12(ks100)* (right) animals expressing GCaMP3 in response to a 30 sec pulse of 10^{-4} hexanol in 10^{-4} sIAA. Video is at 6X speed.

S2 Video. Intracellular calcium levels are increased in AWC^{OFF} upon addition or removal of hexanol in wild-type and *gcy-12* mutants, respectively.

Fluorescence changes in AWC^{OFF} in wild-type (left) and *gcy-12(ks100)* (right) animals in response to a 30 sec pulse of 10^{-4} hexanol in 10^{-4} sIAA. Video is at 6X speed.

bioRxiv preprint doi: https://doi.org/10.1101/2025.07.25.666858; this version posted July 29, 2025. The copyright holder for this preprint (which was not certified by peer review) is the author/funder, who has granted bioRxiv a license to display the preprint in perpetuity. It is made available under aCC-BY 4.0 International license.

S1 Table. Strains used in this work.

Strain	Genotype
WT	N2 (Bristol)
CX3222	*odr-3(n1605)*
CX2065	*odr-1(n1936)*
PY5693	*gcy-12(ks100)*
QD190	*gcy-12(nj10)*
FX02411	*gcy-28(tm2411)*
RB626	*gcy-37(ok384)*
PY12352	*gcy-29(oy183)*
CZ3715	*gcy-33(ok232)*
RB1935	*gcy-20(ok2538)*
AX1297	*gcy-36(db66)*
FX17907	*gcy-9(tm7632)*
RB1048	*gcy-32(ok995)*
RB1909	*gcy-19(ok2472)*
PY10501	*oyIs91[odr-1p::GCaMP3, srsx-3p::mScarlet]*
PY12500	*gcy-12(ks100);oyIs91[odr-1p::GCaMP3, srsx-3p::mScarlet]*
PY12501	*gcy-12(nj10);oyIs91[odr-1p::GCaMP3, srsx-3p::mScarlet]*
PY12502	*oyEx776[odr-1p::gcy-12.a::mStayGold];[odr-1p::gcy-12.b::mScarlet-I3]* Line 1
PY12503	*oyEx777[odr-1p::gcy-12.a::StayGold];[odr-1p::gcy-12.b::mScarlet-I3]* Line 2
PY12504	*loxP::gcy-12::loxP(syb10363);oyIs91[odr-1p::GCaMP3, srsx-3p::mScarlet]*
PY12505	*loxP::gcy-12::loxP(syb10363);oyIs91[odr-1p::GCaMP3, srsx-3p::mScarlet];oyEx778[srsx-3p::Cre::BFP]* Line 1
PY12506	*loxP::gcy-12::loxP(syb10363);oyIs91[odr-1p::GCaMP3, srsx-3p::mScarlet];oyEx779[srsx-3p::Cre::BFP]* Line 2
PY12507	*loxP::gcy-12::loxP(syb10363);oyIs91[odr-1p::GCaMP3, srsx-3p::mScarlet]; oyEx780[odr-1p::Cre::GFP]* Line 1
PY12508	*loxP::gcy-12::loxP(syb10363);oyIs91[odr-1p::GCaMP3, srsx-3p::mScarlet];oyEx781[odr-1p::optCre::BFP]* Line 1
PY12509	*nsy-5(tm1896);oyIs91[odr-1p::GCaMP3, srsx-3p::mScarlet]*
PY12510	*nsy-5(tm1896);gcy-12(ks100);oyIs91[odr-1p::GCaMP3, srsx-3p::mScarlet]*
PY12511	*nsy-1(ok390);oyIs91[odr-1p::GCaMP3, srsx-3p::mScarlet]*
PY12512	*gcy-12(oy213) nsy-1(ok390); oyIs91[odr-1p::GCaMP3, srsx-3p::mScarlet]*
PY12005	*kyIs602[sra-6p::GCaMP3]*
PY12513	*gcy-12(oy212[gcy-12.a::GFP, gcy-12.b::mScarlet-I3])*
PY12514	*loxP::gcy-12::loxP(syb10363);oyIs91[odr-1p::GCaMP3, srsx-3p::mScarlet];oyEx782[srsx-3p::optCre::BFP]* Line 1
PY12515	*loxP::gcy-12::loxP(syb10363);oyIs91[odr-1p::GCaMP3, srsx-3p::mScarlet];oyEx783[srsx-3p::optCre::BFP]* Line 2

bioRxiv preprint doi: https://doi.org/10.1101/2025.07.25.666858; this version posted July 29, 2025. The copyright holder for this preprint (which was not certified by peer review) is the author/funder, who has granted bioRxiv a license to display the preprint in perpetuity. It is made available under aCC-BY 4.0 International license.

S2 Table. Plasmids used in this work.

**Plasmid**	**Description**
PSAB1387	*odr-1*p*::gcy-12.a::mStayGold*
PSAB1388	*odr-1*p*::gcy-12.b::mScarlet-I3*
PSAB1389	*odr-1*p::SV40*NLS-Cre::SL2::NLS-GFP*
PSAB1390	*srsx-3*p::SV40*NLS-Cre::SL2::NLS-BFP*
PSAB1391	*odr-1*p*::egl-13NLS-Cre::SL2::NLS-BFP*
PSAB1392	*srsx-3*p*::egl-13NLS-CRE::SL2::NLS-BFP*

bioRxiv preprint doi: https://doi.org/10.1101/2025.07.25.666858; this version posted July 29, 2025. The copyright holder for this preprint (which was not certified by peer review) is the author/funder, who has granted bioRxiv a license to display the preprint in perpetuity. It is made available under aCC-BY 4.0 International license.

A

Guanylyl cyclases expressed in AWC^{OFF} and AWC^{ON}

Guanylyl cyclase	AWC^{OFF}	AWC^{ON}
daf-11	863	492
odr-1	155	286
gcy-12	12	6
gcy-28	57	68
gcy-37	5	9
gcy-29	120	4
gcy-33	10	38
gcy-20	2	14
gcy-36	-	2
gcy-9	109	44
gcy-32	-	2
gcy-19	15	6

B

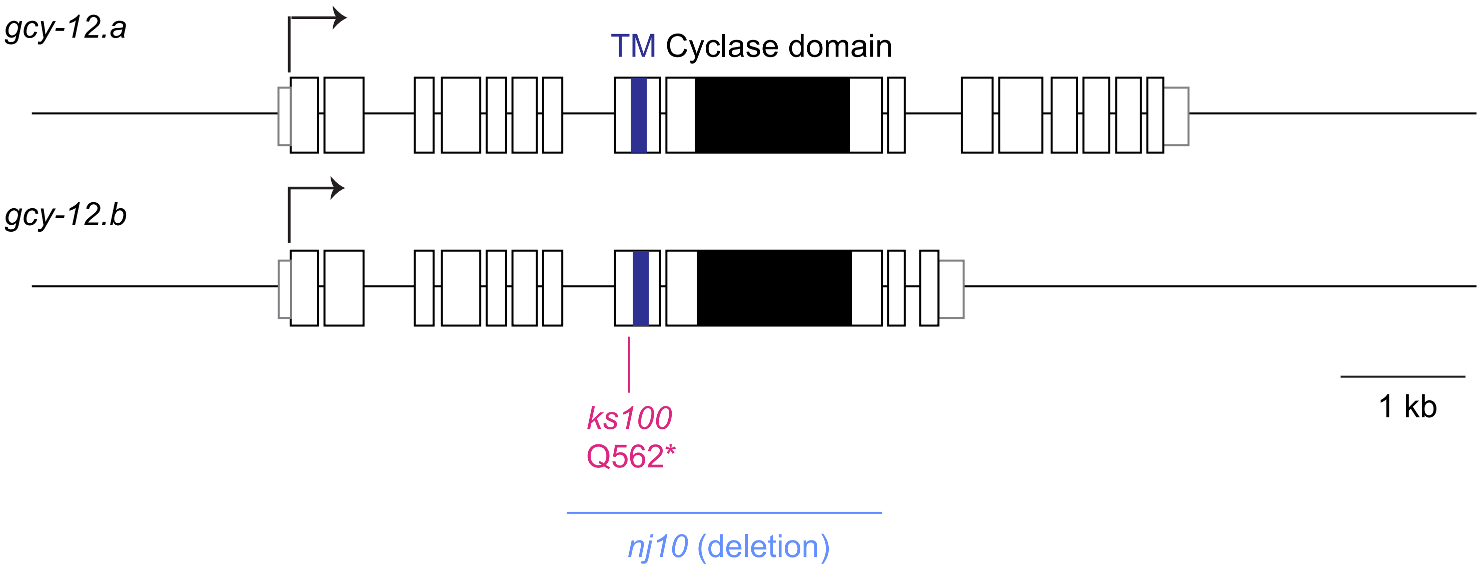

C

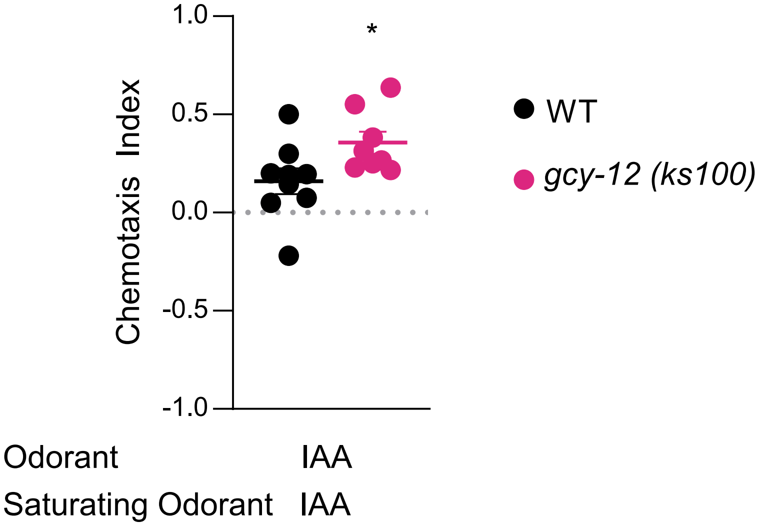

Pandey et al., Figure S1

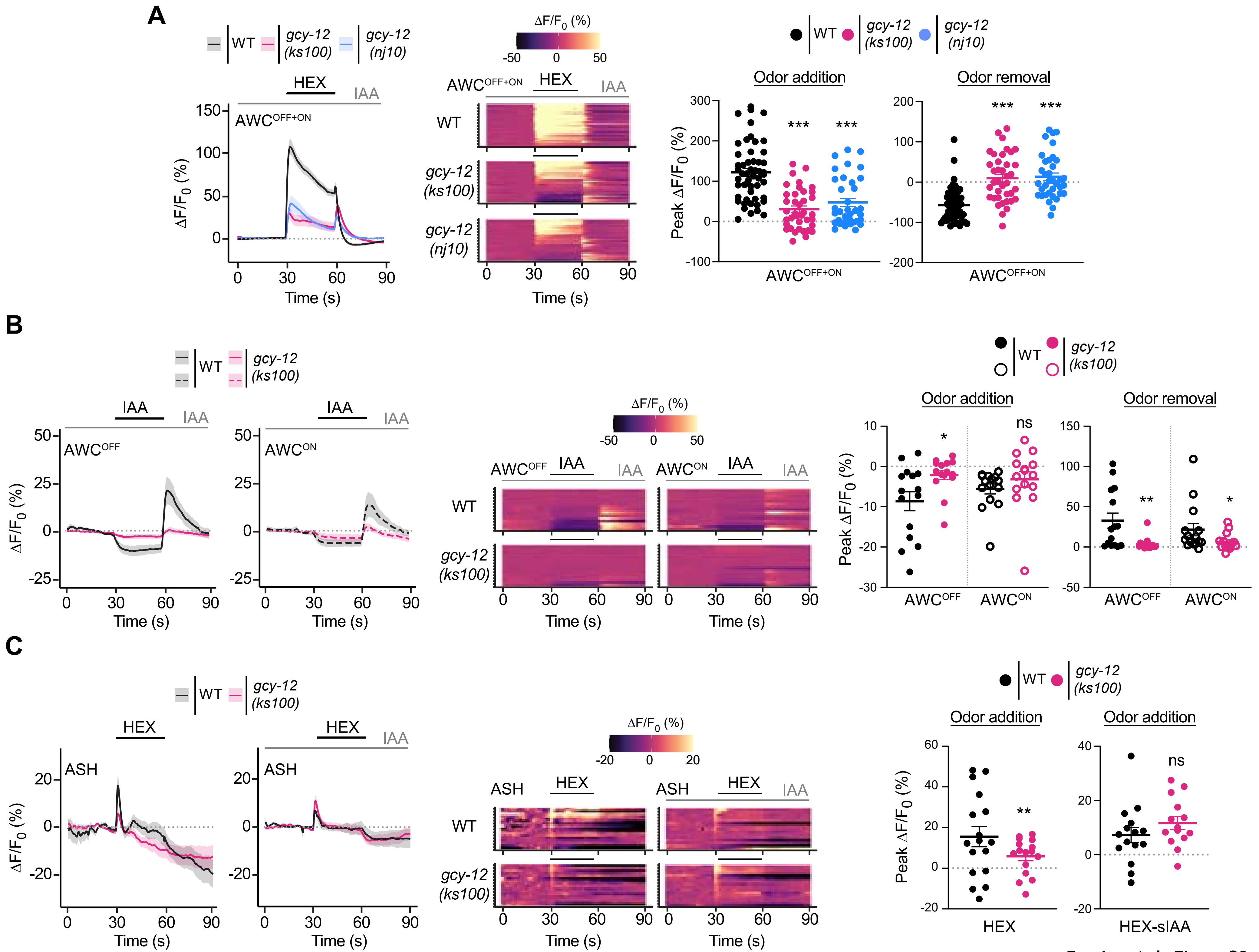

Pandey et al., Figure S2

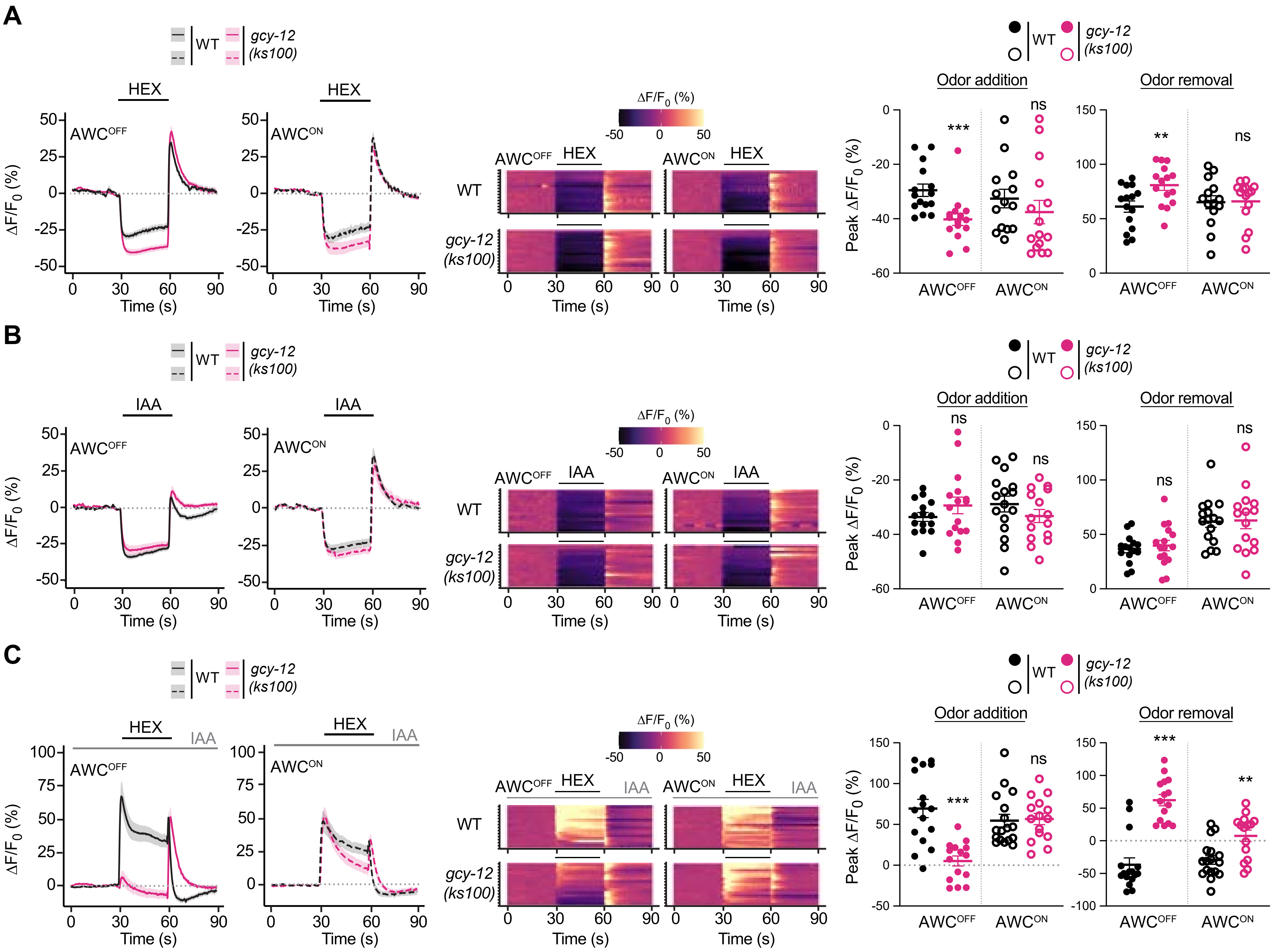

Pandey et al., Figure S3

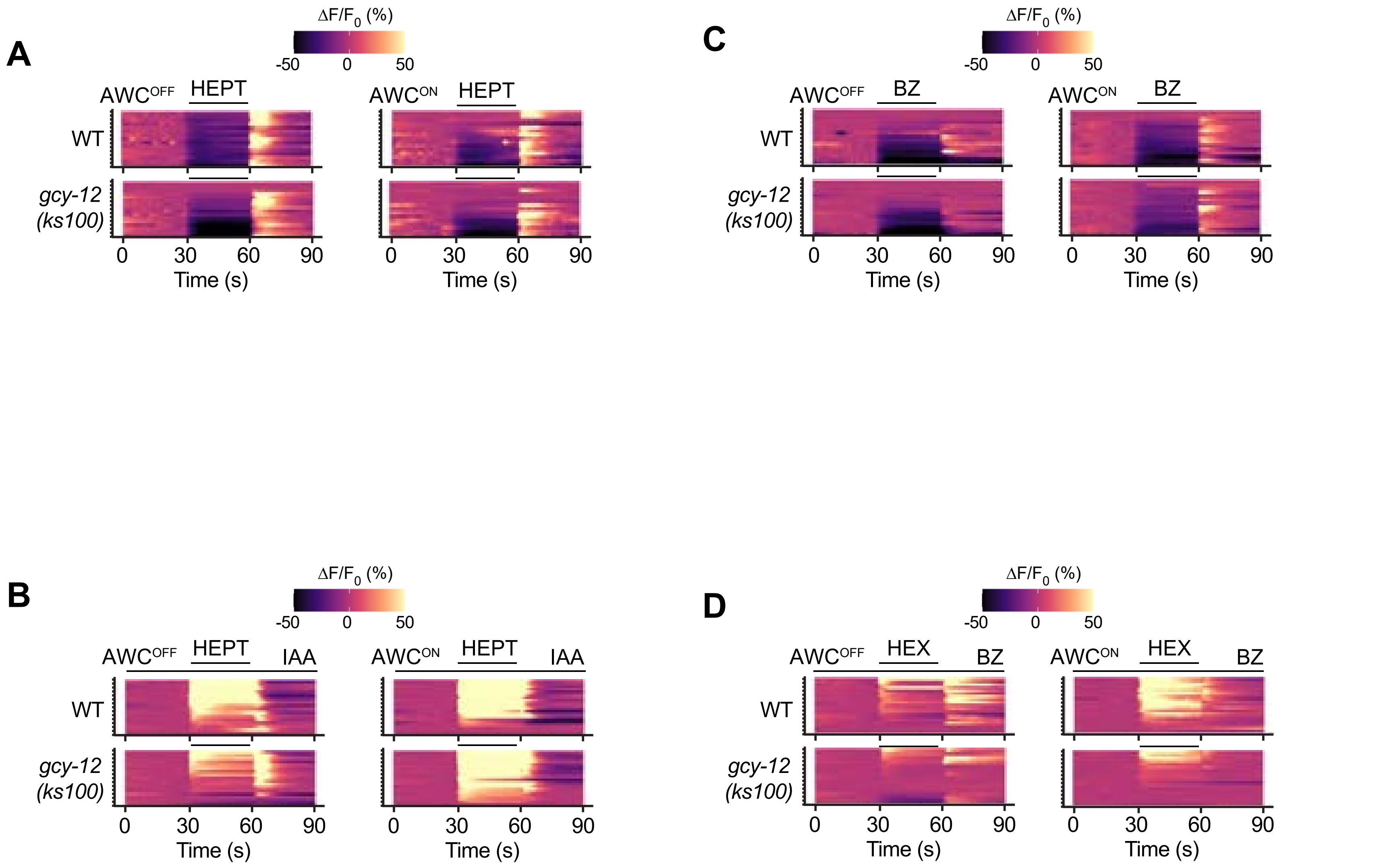
A
B
C
D
ΔF/F0 (%)
-50
0
50
AWCOFF
AWCON
HEPT
IAA
BZ
HEX
WT
gcy-12
(ks100)
0
30
60
90
Time (s)

bioRxiv preprint doi: https://doi.org/10.1101/2025.07.25.666858; this version posted July 29, 2025. The copyright holder for this preprint (which was not certified by peer review) is the author/funder, who has granted bioRxiv a license to display the preprint in perpetuity. It is made available under aCC-BY 4.0 International license.

A

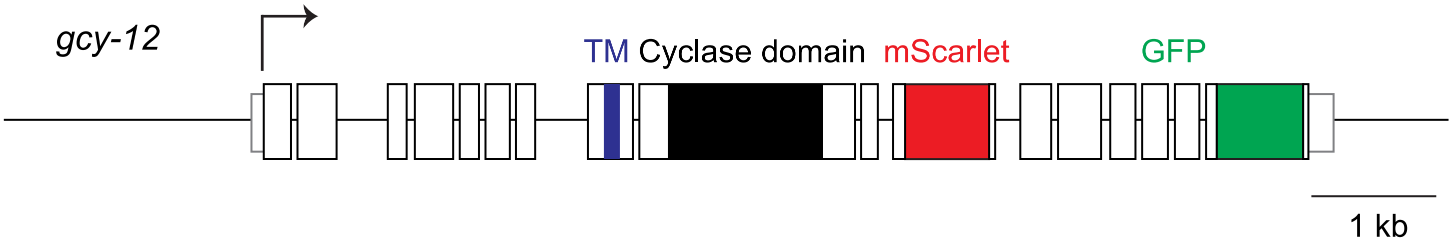

B

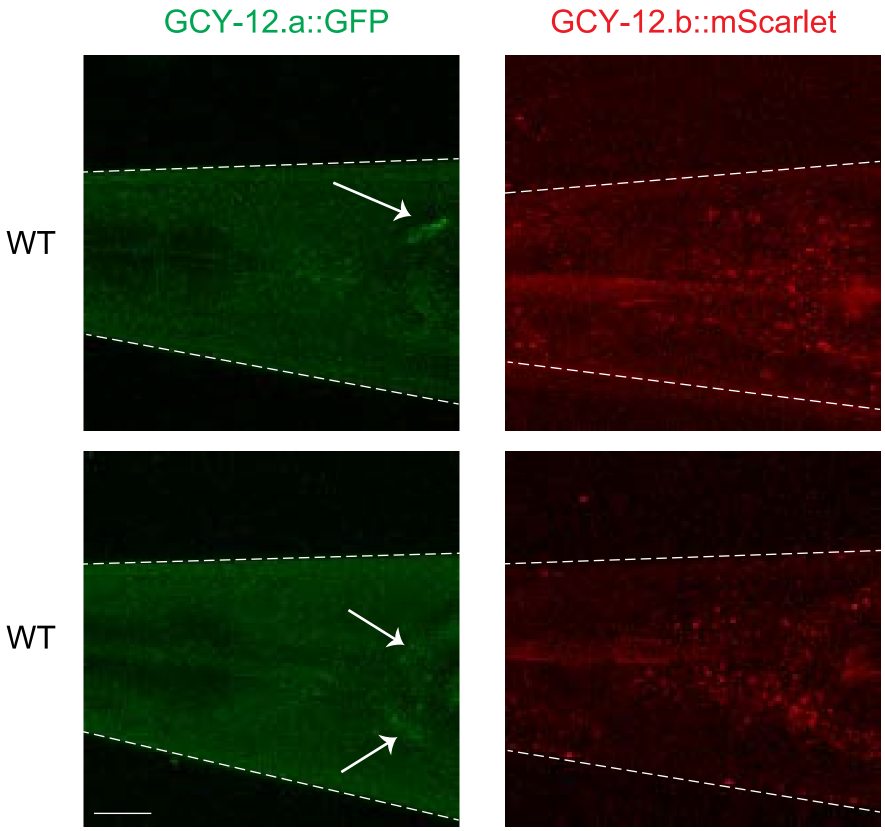

Pandey et al., Figure S5

bioRxiv preprint doi: https://doi.org/10.1101/2025.07.25.666858; this version posted July 29, 2025. The copyright holder for this preprint (which was not certified by peer review) is the author/funder, who has granted bioRxiv a license to display the preprint in perpetuity. It is made available under aCC-BY 4.0 International license.

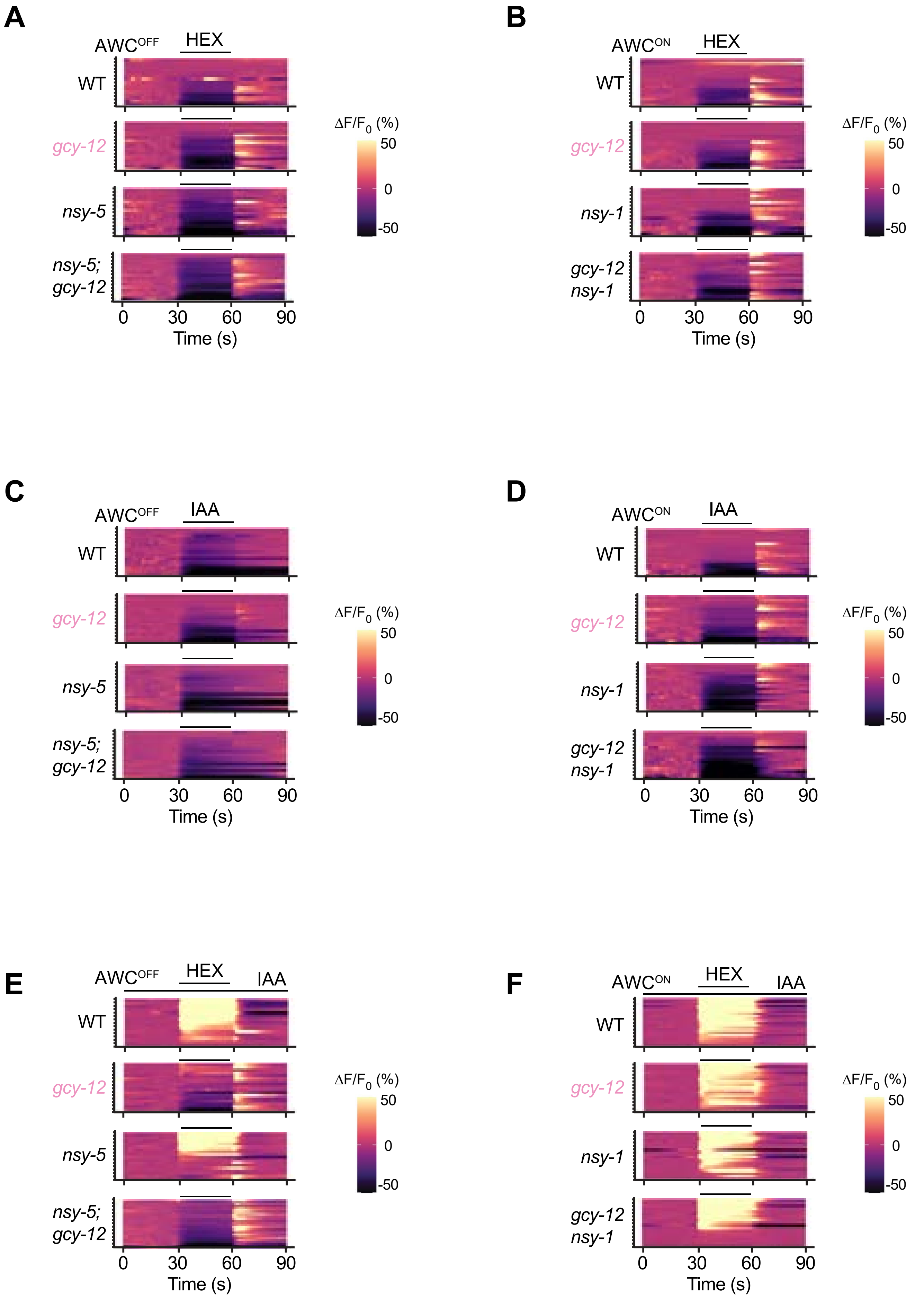

Pandey et al., Figure S6
